# The Relationship between the Structural Characteristics of α-Fe_2_O_3_ Catalysts and Their Lattice Oxygen Reactivity Regarding Hydrogen

**DOI:** 10.3390/ma16124466

**Published:** 2023-06-19

**Authors:** Nadezhda Kirik, Alexander Krylov, Andrey Boronin, Sergey Koshcheev, Leonid Solovyov, Evgenii Rabchevskii, Nina Shishkina, Alexander Anshits

**Affiliations:** 1 Federal Research Center “Krasnoyarsk Science Center of Siberian Branch of the Russian Academy of Sciences”, Institute of Chemistry and Chemical Technology, 50/24, Akademgorodok, 660036 Krasnoyarsk, Russia; leosol@icct.ru (L.S.); rabchev@mail.ru (E.R.); ninash@icct.ru (N.S.); 2 Federal Research Center “Krasnoyarsk Science Center of Siberian Branch of the Russian Academy of Sciences”, Kirensky Institute of Physics, 50/38, Akademgorodok, 660036 Krasnoyarsk, Russia; shusy@iph.krasn.ru; 3 Federal Research Center Boreskov Institute of Catalysis, 5, Ac. Lavrentieva Ave., 630090 Novosibirsk, Russia; boronin@catalysis.ru (A.B.); ser@catalysis.ru (S.K.); 4 Department of Chemistry, 79, Svobodny Ave., Siberian Federal University, 660041 Krasnoyarsk, Russia

**Keywords:** α-Fe_2_O_3_, catalysts, calcinations, XRD, XPS, Raman spectroscopy characterization, temperature-programmed reduction

## Abstract

In this paper, the relationship between the structural features of hematite samples calcined in the interval of 800–1100 °C and their reactivity regarding hydrogen studied in the temperature-programmed reaction (TPR-H_2_) was studied. The oxygen reactivity of the samples decreases with the increasing calcination temperature. The study of calcined hematite samples used X-ray Diffraction (XRD), Scanning Electron Microscopy (SEM), X-ray Photoelectron Spectroscopy (XPS), and Raman spectroscopy, and their textural characteristics were studied also. According to XRD results, hematite samples calcined in the temperature range under study are monophase, represented by the α-Fe_2_O_3_ phase, in which crystal density increases with increasing calcination temperature. The Raman spectroscopy results also register only the α-Fe_2_O_3_ phase; the samples consist of large, well-crystallized particles with smaller particles on their surface, having a significantly lower degree of crystallinity, and their proportion decreases with increasing calcination temperature. XPS results show the α-Fe_2_O_3_ surface enriched with Fe^2+^ ions, whose proportion increases with increasing calcination temperature, which leads to an increase in the lattice oxygen binding energy and a decrease in the α-Fe_2_O_3_ reactivity regarding hydrogen.

## 1. Introduction

Iron oxides are actively studied as catalysts in oxidation processes and as oxygen carriers in high-temperature chemical lopping processes, such as carbonaceous fuels combustion (CLC), reforming (CLR), hydrogen generation (CLGH), and partial methane oxidation (CLPO) [1,2,3,4,5,6,7]. Compared with other oxide systems, iron oxide-based materials have several advantages: the main ones are the possibility of modifying the reactivity, high oxygen capacity, high melting temperatures, mechanical strength characteristics, low cost, and natural compatibility.

High-temperature pretreatment of iron oxide-based catalysts affects not only the specific surface area, crystallite sizes, and mechanical properties, but also influences the surface atomic structure and structural characteristics of the oxides [8,9]. In particular, the temperature treatment of nanosized hematite samples in the range of 250–900 °C demonstrated the areas of structural disorder in the crystal lattice connected with the transformation of octahedral Fe^3+^ positions into tetrahedral Fe^2+^ ones after sample calcination at 900 °C [8]. During heat treatment of synthetic goethite up to 1100 °C, the formation of stoichiometric hematite after calcination at 500 °C occurs. For the structure of samples calcined up to 800 °C, the presence of flat defects in the basal plane is characteristic; with further increase in temperature, these defects disappear, and the formation of extended defects occurs, such as twins and surface steps, stabilizing in high-temperature (900–1100 °C) samples due to impurities [9].

The effect of high-temperature pretreatment (850–950 °C) of ore with a hematite content of 95 wt.% on the stabilization of activity during methane conversion in the CLC process is shown in [10]. The activity of calcined samples is maintained for 80 consecutive red-ox cycles, which is an important characteristic of oxygen carriers in cyclic processes.

In high-temperature CLC processes, at the first stage (in a fuel reactor) the fuel interacts with the oxygen carrier in the oxidized form [11,12,13], and the oxidation of hydrocarbons, hydrogen, and/or carbon monoxide occurs due to the lattice oxygen of the oxide, while the oxide crystal lattice at the initial stage transforms with the formation of surface vacancies in the anion sublattice [5,14,15]. The completely reduced oxygen carrier is then oxidized at the second stage (in an air or steam reactor) [11,12,13].

The high-temperature effect of obtaining hematite samples with α-Fe_2_O_3_ structure on the reactivity regarding hydrogen is found in several works [16,17,18,19]. With increasing calcination temperature in the range of 500–1200 °C of hematite samples obtained from different precursors, the initial reduction temperature (T_in_) in the temperature-programmed reaction (TPR), in general, increases [16]. Samples synthesized from different precursors have different T_in_ after calcination at a fixed temperature. The difference also remains after high-temperature calcination (1100–1200 °C), which apparently relates to the differences in bulk and surface properties of α-Fe_2_O_3_ structure. It was also shown that the values of the activation energy vary during the reduction in the hydrogen of hematite samples with heat treatment (1200 °C) and without it [18].

Thus, the temperature pretreatment of α-Fe_2_O_3_ and its oxide-based systems is an essential factor affecting the red-ox properties, reducibility of oxides, and their reactivity to reducing agents (hydrogen, carbon monoxide, methane). Complex studies of the relationship between the calcination temperature, structural characteristics, and hematite oxidative reactivity have not been practically conducted. Previously, we showed [19] that increasing the calcination temperature of hematite samples with corundum structure in the range of 800–1100 °C leads to a consistent increase in the crystal density of the α-Fe_2_O_3_ phase, indicating a decrease in the degree of crystal lattice disorder and the growth of lattice oxygen binding energy, which manifests itself in a significant decrease in the α-Fe_2_O_3_ reactivity regarding hydrogen. The present work aimed to investigate the relationship between the structural features, surface state, and the lattice oxygen reactivity regarding H_2_ (as model reducing agent) of the α-Fe_2_O_3_ samples obtained using high-temperature (800–1100 °C) synthesis.

## 2. Materials and Methods

### 2.1. Chemicals and Materials

To prepare samples of α-Fe_2_O_3_, we used a powdered reagent of Fe_2_O_3_ (TC 6-09-1418-78, high purity qualification, heavy metal (Cu, Co, Sb) impurities < 5.0·10^−3^ wt. %). The reagent was dried at 200 °C for 2 h to remove hygroscopic moisture. The dried and fractionated material (˂40 μm), weighing 0.7–1.0 g, was pressed at 390 MPa with a holding time of 4 min into tablets of 16 mm diameter, about 1.0–1.5 mm thick, then calcined in air at 800, 900, 1000 and 1100 °C for 10 h and cooled to room temperature at a rate of ~8°/min. Sintered samples were milled, alternating crushing stages in a ball mill KM-1 (VEB, DDR) for 5 min and sieving on a vibratory drive VP/S-220 (Vibrotechnik, Russia) for 2 min, with selecting fractions of particle size class −0.2 + 0.1 mm. Marking of the samples used the values of calcination temperature; for example, sample Fe_2_O_3_-800 was calcined at 800 °C.

### 2.2. Characterization Techniques

The measurement of the texture characteristics of the obtained samples used a NOVA 3200e sorption analyzer (Quantachrome Instruments, USA) in the low-temperature nitrogen adsorption mode at −195.8 °C with samples degassed at 300 °C for 12 h to a residual pressure of <10^−2^ mm Hg. The specific surface area (SSA) calculation used a modified BET method [20,21], which makes it possible to consider the presence of micropores in the meso-macroporous material. The volume of micropores (V_mic_) and mesopores (V_meso_) was analyzed by the comparative t-method using the de Boer equation to calculate the thickness of the statistical adsorbate layer [22]. The specific surface area and pore volume were averaged using the values of 4 independent measurements.

Morphologies of calcined hematite samples were studied using scanning electron microscopy (SEM) with a TM-4000 (Hitachi, Tokyo, Japan) instrument.

X-ray diffraction patterns were recorded on an X’Pert Pro MPD diffractometer (PANalytical, Almelo, The Netherlands) with a high-speed PIXcel detector in the angular interval of 23–153° 2Θ, in steps of 0.013° on Cu K_α_ radiation. The crystal lattice parameters of the hematite phase were determined and refined using the Rietveld full-profile approach using the derivative difference minimization (DDM) method [23,24,25], the XRD peak shift due to the sample surface misalignment was included in the Rietveld full-profile model and the respective displacement parameter was refined along with the lattice parameters to minimize the systematic errors. Owing to well-resolved diffraction peaks in the wide diffraction angle interval used, the lattice and peak shift parameters were correctly and precisely refined.

The Raman spectra were recorded in the wavenumbers range of 50–1600 cm^−1^ in the backscattering configuration on a T64000 triple spectrometer (HORIBA Jobin Yvon, Palaiseau, France) with a spectral resolution of 2 cm^−1^. To excite the sample, a diode-pumped solid-state Excelsior-532-300-CDRH laser (Spectra-Physics, Milpitas, CA, USA) with a wavelength of 532 nm and a power of 1 mW was used on the sample. The spectra were obtained in an air atmosphere from the sample located on a glass slide. The excitation radiation was focused on the sample using an Olympus BX41 microscope through a 50× Olympus MPlan objective with a numerical aperture of N.A = 0.75 into a spot 3 µm in diameter.

### 2.3. Examination of the State Surface of Calcined α-Fe_2_O_3_ Samples

The surface state of calcined hematite samples was examined using X-ray photoelectron spectroscopy on an ES300 photoelectron spectrometer (KRATOS Analytical, Manchester, UK) calibrated along Au*4f*_7/2_ and Cu*2p*_3/2_ lines with 84.0 and 932.7 eV binding energies for gold and copper metal foils, respectively [26]. A nonmonochromatized Mg Kα X-ray source with 1253.6 eV energy was used to record the spectra. To exclude the sample charging effect, the spectra were calibrated by shifting all recorded spectral lines by the value corresponding to the electrostatic charge using the C*1s* reference peak from amorphous carbon with E_b_(C*1s*) = 285.0 eV. In the investigated samples, this value of E_b_(C*1s*) plays the role of being an internal standard for binding energies of lines of other elements. A conductive adhesive tape specifically used for vacuum technologies provided the electrical contact of the samples with the metal holder. All samples were carefully grounded in an agate mortar before taping.

For the identification of lines and qualitative elemental composition of the surface, the survey spectrum in the range of 0–1200 eV was used. The detailed high-resolution spectra of lines Fe*2p*, O*1s*, C*1s*, etc. were registered to analyze the quantitative composition of samples and the chemical surface state. Calculations of sample composition were performed with the help of atomic sensitivity factors (ASF) [27,28].

Spectra were decomposed into individual components using the Gauss and Lorentz functions after subtracting the scattered electron background using the Shirley method. The spectra processing was performed using the original XPSCalc Software tested on oxide [29] and metal-oxide systems [30].

### 2.4. Temperature-Programmed Reduction of α-Fe_2_O_3_ Samples by Hydrogen

Temperature-programmed reduction measurements were carried out in a TG-DSC NETZSCH STA 449C (Germany) analyzer equipped with an Aeolos QMS 403C mass spectrometer, under 5%H_2_ + 95%Ar (H_2_ grade A, 99.99 vol.%, Ar grade 5.0, 99.999 vol.%) atmosphere at ambient pressure with heating in the range of 40–900 °C in Pt crucibles without lids (30.00 ± 0.01 mg, β = 5°/min, total flow 100 cm^3^/min). The qualitative composition of the gas phase was determined using on-line QMS in multiple ion detection mode from the intensity of ions of *m*/*z* = 18 (H_2_O), 32 (O_2_), 44 (CO_2_), and 28 (CO). Calibration of the sensor sensitivity by heat flow was performed by measuring the heat capacity of a standard sapphire disk using the method in [31]. The reduction degree of hematite was calculated from the mass loss with the recalculation of the removed lattice oxygen amount, the degree of 100% reduction corresponds to the oxygen removal from the lattice α-Fe_2_O_3_ when reduced to metal, the degrees of 11.1 and 33.3% reduction correspond to the reduction of hematite to magnetite and wüstite.

## 3. Results and Discussion

### 3.1. Reactivity of Calcined α-Fe_2_O_3_ Samples in the Temperature-Programmed Reduction by Hydrogen

The reactivity of monophase samples α-Fe_2_O_3_ preheated at 800–1100 °C significantly decreases with increasing treatment temperature in temperature-programmed reduction by hydrogen (TPR-H_2_). Table 1 shows the physicochemical characteristics of the calcined samples (specific surface area SSA, total pore volume V_pore_, average particle size D_av_, and crystal density *D_X_*) and the values of initial reduction temperature (T_in_) and temperature of reaching 95% reduction degree (T_95%_). According to Table 1, the sample calcined at 800 °C shows the most activity and has the lowest temperatures of T_in_ and T_95%_.

**Figure 1:**
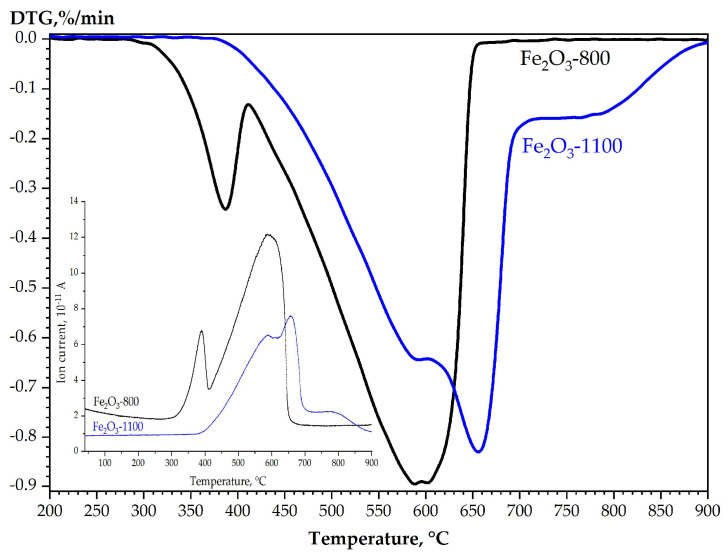
TPR profiles of samples Fe_2_O_3_-800 and Fe_2_O_3_-1100: DTG curves and MS (*m*/*z* = 18) curves in the insert.

The calcination temperature of α-Fe_2_O_3_ also determines the staging of the reduction process. Figure 1 shows the differential thermo-gravimetric (DTG) and water release (molecular ion mass spectrum with *m*/*z* = 18) curves during the reduction of Fe_2_O_3_-800 and Fe_2_O_3_-1100 samples.

The low-temperature peaks on the DTG and mass spectra (*m*/*z* = 18) curves (Figure 1) during the reduction of the Fe_2_O_3_-800 sample correspond to the stage of magnetite formation, confirmed by XRD results, according to which, after the reduction at the temperature up to 410 °C, the sample contains 98.8% of Fe_3_O_4_ and 1.2% of α-Fe. The second broad peak corresponds to the subsequent combined stages of oxides reduction to metal. Increasing the calcination temperature leads to forming of α-Fe_2_O_3_ samples whose reactivity regarding hydrogen significantly decreases; the reduction process of α-Fe_2_O_3_ begins at temperatures 50–100 °C higher (Table 1). The reduction of α-Fe_2_O_3_ samples calcined at 1000–1100 °C occurs without the magnetite formation stage and does not end at 900 °C (Figure 1).

It is known that it is possible to change the oxidizing ability of transition metal oxides by activating the Me-O bond. One of the methods is the modification of oxide by other cations to create a defective structure [32]. In our work, we used hematite samples from high-purity qualification reagents, and a change in their reactivity with respect to hydrogen is associated with structural features that are due to the temperature factor during the synthesis of samples. Considering that the hematite samples calcined at 800–1100 °C are monophase, we may assume that the decrease in oxygen reactivity regarding hydrogen is associated with the increase in lattice oxygen binding energy due to forming more ordered bulk crystal lattice and surface changes with increasing calcination temperature of α-Fe_2_O_3_.

To reveal the effect of calcination temperature of monophase α-Fe_2_O_3_ samples on the degree of disorder/violation of the near-order in the crystal lattice and the surface chemical state, the samples calcined in the range of 800–1100 °C were investigated using Raman spectroscopy and XPS.

### 3.2. Examination of Calcined α-Fe_2_O_3_ Samples using Raman Spectroscopy

**Figure 2:**
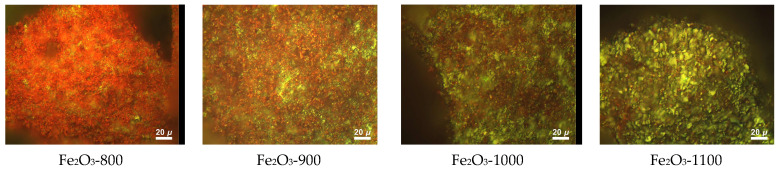
Images of α-Fe_2_O_3_ samples calcined in the range of 800–1100 °C.

Figure 2 shows images of α-Fe_2_O_3_ samples grains, calcined in the interval of 800–1100 °C, obtained with a microscope attachment in the Raman spectrometer. All samples are represented by particles of two types distinguishable by color under imaging conditions with assigned color codes “red” and “green.” Here, the ratio of particles changes with the calcination temperature. Smaller “red” particles practically completely cover the particles of the low-temperature Fe_2_O_3_-800 sample, under which one can see “green” particles.

**Figure 3:**
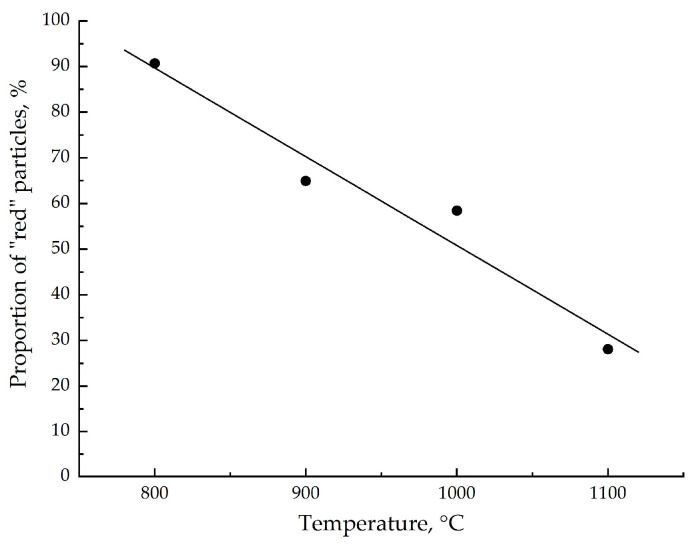
The proportion of surface “red” particles in dependency of α-Fe_2_O_3_ calcination temperature.

**Figure 4:**
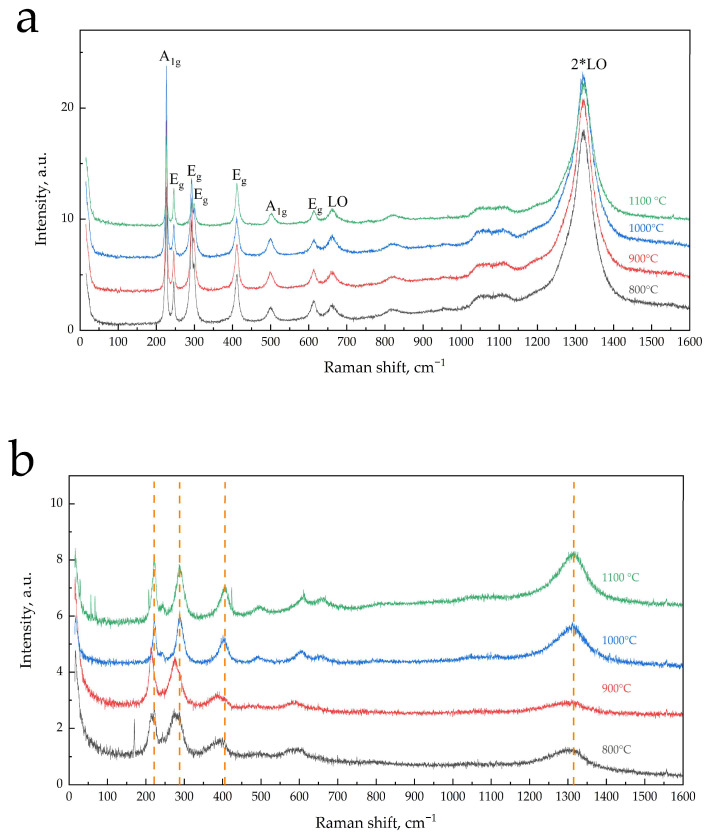
Raman spectra of calcined (800–1100 °C) α-Fe_2_O_3_ samples, in the range of 50–1600 cm^−1^: (**a**) spectra of “green” particles with the phonon peak assignments and (**b**) spectra of “red” particles.

As the calcination temperature increases, the size of “red” particles increases (Figure 2), their contribution significantly decreases (Figure 3), and mainly “green” particles represent the high-temperature Fe_2_O_3_-1100 sample. The proportion of surface “red” particles was calculated as the area of red regions relative to the sum of the red and green regions from the presented images. We can assume that the presence of two types of particles is due to different types of disorder in the crystal lattice of α-Fe_2_O_3_. To clarify the situation, we analyzed the Raman spectra taken from the “green” and “red” particles in the calcined samples of α-Fe_2_O_3_ (Figure 4).

The spectra of “green” and “red” particles are close, which indicates phase homogeneity, but as seen from Figure 4a,b, lines in the spectra significantly differ in intensity and magnitude of FWHM (full widths at half maximum), indicating a significant difference in the degree of crystallinity phase in particles. According to the literature data, all registered peaks are characteristic of α-Fe_2_O_3_. Thus, the calculated Raman spectrum of α-Fe_2_O_3_ in the region up to 700 cm^−1^ includes seven phonon lines related to transverse (TO) vibrational modes, two A_1g_ modes, and five E_g_ modes [33,34], which correspond to peaks recorded, respectively, at 225–229 cm^−1^ and 498–500 cm^−1^ and at 245–249, 293–295, 298–302, 412–414, and 612–615 cm^−1^ in the experimental spectra [33,34,35,36]. In the experimental spectra of powder α-Fe_2_O_3_ samples, besides TO-mode lines, the lines belonging to the longitudinal (LO) modes are also registered at 660 and 1320 cm^−1^, respectively [33,35,36,37].

**Figure 5:**
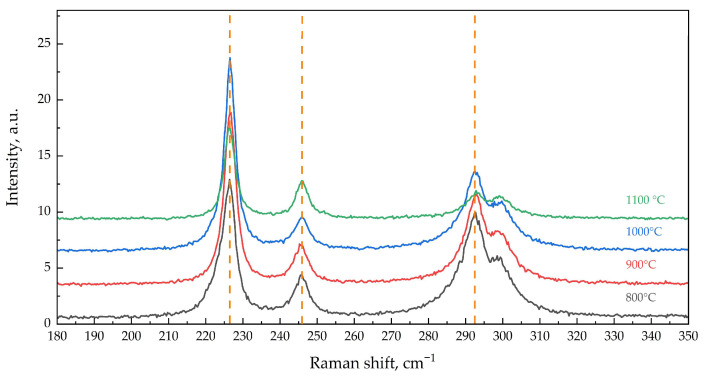
Raman spectra of “green” particles, in the range of 180–350 cm^−1^.

As seen from the spectra of “green” particles shown in Figure 4a, all calculated TO and LO lines of α-Fe_2_O_3_ are present with good resolution in all calcined samples in the range of 50–1600 cm^−1^. The spectrum of Fe_2_O_3_-800 contains lines (cm^−1^) at 226 (A_1g_), 246 (E_g_), 293 (E_g_), shoulder at 299 (E_g_), 412 (E_g_), 497 (A_1g_), 614 (E_g_), 659 (LO), and 1318 (2*LO). Here, with increasing the calcination temperature of α-Fe_2_O_3_, the line positions do not change (Figure 4a, 5); however, changes in line intensities (cm^−1^) at 226 (A_1g_), 293 (E_g_), and the shoulder at 299 (E_g_) occur. Small, up to 1.3 times, variations in the intensities of the remaining lines are apparently due to the unequal contribution of different crystal planes on the surface and/or surface roughness [33,36]. In the spectra of “green” particles of low-temperature (800–900 °C) α-Fe_2_O_3_ samples, there is an asymmetric broadening of the peaks clearly visible for the peak at 226 cm^−1^ in the spectrum of the Fe_2_O_3_-800 sample (Figure 5). The asymmetry of the peaks practically disappears after the calcination of the samples at 1000–1100 °C. The broadening of the peaks may be due to the size factor observed in the Raman spectra of nanosized particles of α-Fe_2_O_3_ in film samples [36].

In the Raman spectra of α-Fe_2_O_3_, the line at 225–229 cm^−1^ (A_1g_) belongs to the vibrations of iron ions along the *c* crystallographic axis [35]. Calculation from X-ray diffraction data of the parameter *c* of the unit cell of α-Fe_2_O_3_ calcined samples showed a decrease from 13.7472(1) to 13.7416(1) Å when increasing the calcination temperature from 800 to 1000 °C, and further showed an increase to 13.7438(1) after calcination at 1100 °C [20], which is in inverse correlation with the change in line intensity at 226 cm^−1^. The ratio of this line intensity to the slightly variable line intensity at 246 cm^−1^, I_226_ cm^−1^/I_246_ cm^−1^, increases from 3.4 to 6 with increasing calcination temperature to 1000 °C and decreases to 2.5 after calcination at 1100 °C. The line at 293 cm^−1^ (E_g_) refers to one of the two external vibrations [33]. Its decrease in intensity with increasing calcination temperature of α-Fe_2_O_3_ (Figure 5) probably relates to the decrease in surface/volume ratio with increasing crystallite size.

It is worth noting that in the spectra of “green” particles of all investigated α-Fe_2_O_3_ samples, the LO mode line at 659 cm^−1^ is registered (Figure 4a). This mode is prohibited in the Raman spectrum of α-Fe_2_O_3_, and the activation of this mode, as noted in several studies, is due to the presence of structural disorder/defectivity in the crystal lattice [33,35,36,37]. Note that in the 667–670 cm^−1^ region, the magnetite phase also has the Raman line (A_1g_) [36,37,38]. However, the authors of [37], based on comparing the spectra of the hematite and mixed magnetite with hematite samples, unambiguously attribute this line to the LO hematite mode activated due to the structural disorder. Considering the above data and the monophasic nature of the calcined samples α-Fe_2_O_3_ (by XRD), we can argue that the peak at 659 cm^−1^ in the Raman spectra of “green” particles of all samples is due to the disorder in the α-Fe_2_O_3_ crystal lattice. Registration in the spectra of an intense line at 1318 cm^−1^ (Figure 4a) (double the value of the LO mode wave number), which refers to the vibrational mode 2*LO, is consistent with the results of [33,38], in which the study of hematite samples also used green laser (514.5, 532.2 nm) for excitation and this intense line was recorded. The authors associate the significant intensity of this line with the resonance excitation effect.

The Raman spectra analysis of “red” particles (Figure 4b), as already mentioned, shows their correspondence also to the spectrum of α-Fe_2_O_3_ oxide, while there are features compared with the spectra of α-Fe_2_O_3_ “green” particles. All peaks have lower ~8–10-fold intensity and higher ~3–5-fold FWHM values, unresolved peaks in the 290 cm^−1^ region, and an observed shift of all peaks to lower values of wave numbers for high-temperature samples (except for the line at 662 cm^−1^) by 5–10 cm^−1^ and for low-temperature samples by 15–25 cm^−1^. The peak at 662 cm^−1^ attracts attention, being registered only in the spectra of high-temperature samples with the position shifted toward higher wave number values, unlike the other lines. Perhaps, the peak at 662 cm^−1^ is a superposition of the LO mode of hematite and the most intense in this region of the magnetite mode (A_1g_). The LO mode wave number of hematite based on 1306 cm^−1^ for the 2LO mode must have the value of 653 cm^−1^, i.e., the LO peak, as well as other peaks in the spectra of α-Fe_2_O_3_ “red” particles of high-temperature samples, is shifted to lower values of the wave number compared with the peaks of α-Fe_2_O_3_ “green” particles.

Thus, the study of α-Fe_2_O_3_ samples calcined in the interval of 800–1100 °C using Raman spectroscopy allows us to draw the following conclusions. All samples consist of two types of particles represented by the α-Fe_2_O_3_ phase differing in crystallite size, degree of crystallinity, and presence of disorder in the crystal lattice. “Red” particles that are smaller are located on the surface of larger “green” ones. In large particles, the α-Fe_2_O_3_ phase is well crystallized, and there is a disorder in the crystal lattice, as evidenced by the LO mode at 659 cm^−1^. Surface particles also consist of the α-Fe_2_O_3_ phase with a lower degree of crystallinity and smaller crystallites; the size of the particles increases with increasing sample calcination temperature with an overall decrease in their content. In the surface particles of high-temperature (1000–1100 °C) samples, along with the phase of α-Fe_2_O_3_, which has a disorder in the crystal lattice similar to large particles, traces of the magnetite phase are also possible.

### 3.3. Study of the α-Fe_2_O_3_ Surface in Calcined Samples

The influence of the calcination temperature on the surface state of α-Fe_2_O_3_ was also investigated using the XPS method. Analysis of the survey spectra lines in the 0–1150 eV range showed primary (Fe, O) and impurity (C, Si) elements, Si appeared due to the procedure of grinding sintered samples (see the footer Table 2) and C was revealed to be present as elementary carbon and carbonate species on the surface of the sample. Table 2 shows the calculated surface composition of the calcined samples of α-Fe_2_O_3_.

The valence states of the elements were estimated from the XPS spectra on the base of the peak position in the spectrum together with the elastic peak/satellite combination [39,40,41,42,43]. For this purpose, we decomposed the integral lines into elementary doublets in the case of Fe*2p* spectra and into individual singlet peaks in the case of O*1s* and C*1s* spectra. Considering the Raman results, we decomposed the Fe*2p* spectra using the literature data on the positions of the main doublets and their shake-up satellites for the Fe^2+^ and Fe^3+^ states, i.e., two doublets from Fe^2+^ with E_b_(Fe*2p*_3/2_)~710 and 714 eV and two doublets from Fe^3+^ with E_b_(Fe*2p*_3/2_)~711.5 and 719 eV. Table 3 shows the positions of the elementary components of the Fe*2p* spectra, which agree well with the literature data [39,40,41,42].

**Figure 6:**
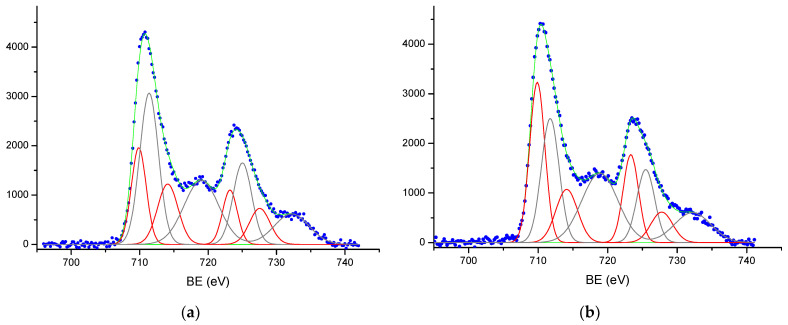
The XPS spectra of Fe*2p* from the surface of hematite samples and the results of curve fitting procedure: (**a**) Fe_2_O_3_-900 and (**b**) Fe_2_O_3_-1100. The experimental data are shown as blue dots, the results of the spectra fitting are shown as solid green lines, the Fe*2p* spectra were decomposed with two doublets from Fe^2+^ (red lines) and two doublets from Fe^3+^ (black lines).

Figure 6 shows, as an example, the Fe*2p* spectra with their decomposition for samples Fe_2_O_3_-900 and Fe_2_O_3_-1100. Analysis of the Fe*2p* spectra showed that the characteristics of Fe^2+^ and Fe^3+^ ions are clearly visible in spectra for all samples. The calculation of the surface composition of hematite samples calcined at 800–1100 °C indicated the deviation from the stoichiometric composition of the Fe_2_O_3_ phase. The ratio of the integral intensity of doublets associated with the Fe^2+^ state to the total area of all doublets of the Fe*2p* line determined the proportion of Fe^2+^ ions on the surface of calcined hematite samples. In samples calcined at temperatures of 800–900 °C, the Fe^2+^/Fe ratio was 0.36–0.38 (0.37–0.42 at 1000–1100 °C), which is higher than the value of 0.33 for the stoichiometric composition of Fe_3_O_4_ oxide.

Knowing that iron oxide with the α-Fe_2_O_3_ structure has high structural stability, at high temperatures, there is a slight deviation from oxygen stoichiometry, and the hematite lattice is oxygen deficient: α-Fe_2_O_3-ε_, where ε is a positive value due to the preferential formation of anion vacancies [44,45]. At 1100 °C, ε is 2⋅10^−5^, and at 1300 °C—7.73⋅10^−4^, respectively, at lgP_O2_ (atm.) = −0.843 and −0.846 [44]. Compared with the bulk, the concentration of anionic vacancies in the surface/subsurface layers of hematite is higher [46], and the deviation from stoichiometry becomes present already at temperatures above 600 °C [47].

Based on the above data, the reason for the observed partial reduction in the surface/subsurface layers of α-Fe_2_O_3_ after calcination at temperatures of 800–1100 °C and the increase in the content of Fe^2+^ ions with increasing temperature is the process of removing oxygen from the α-Fe_2_O_3_ lattice, which leads to the formation of anion vacancies and stabilization of Fe^2+^ cations due to localization of electrons at Fe*3d* levels [45,48,49].

**Figure 7:**
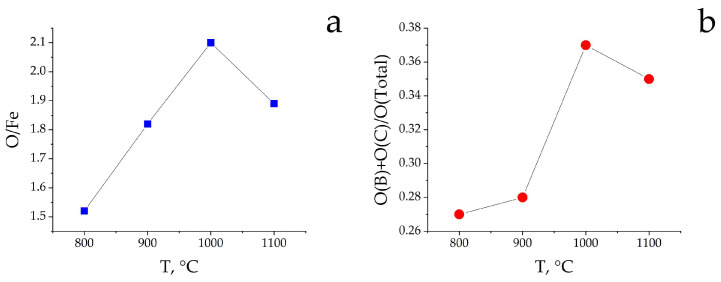
Dependence of ratios of (**a**) O/Fe and (**b**) O(B)+O(C)/O on the α-Fe_2_O_3_ calcination temperature.

Enriching the surface of α-Fe_2_O_3_ calcined samples with low-valent iron ions should decrease the surface O/Fe ratio. However, the evaluation from XPS showed that the O/Fe ratio (when calculating the pure O/Fe ratio, the stoichiometry of O/Si was taken into account) on the surface of calcined samples increases with increasing calcination temperature up to 1000 °C, then slightly decreases (Figure 7a). Only for the low-temperature Fe_2_O_3_-800 sample, the O/Fe value is close to the composition of Fe_2_O_3_, for the rest samples, it is much higher.

**Figure 8:**
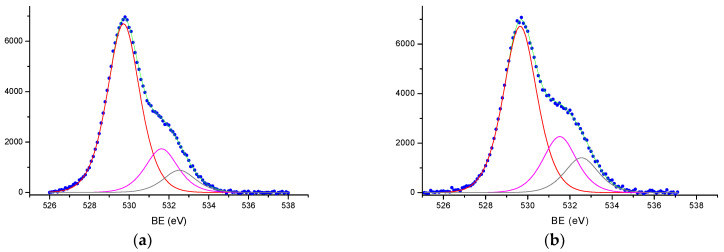
The XPS spectra of O*1s* from the surface of hematite samples and the results of curve fitting procedure: (**a**) Fe_2_O_3_-900 and (**b**) Fe_2_O_3_-1100. The experimental data are shown as blue dots, the results of the spectra fitting are shown as solid green lines, the O*1s* spectra were decomposed with three elementary peaks (red, crimson and black lines).

The obtained O/Fe values testify to the presence of other oxygen forms in addition to the lattice oxygen, also confirmed by the complex shape of the O*1s* peak with a pronounced shoulder on the side of higher binding energies (Figure 8). To determine the fraction of out-of-lattice oxygen forms, the integral O*1s* spectra were decomposed into their components. Figure 8 shows the decomposition of the O*1s* XPS spectra for samples Fe_2_O_3_-900 and Fe_2_O_3_-1100. Here, the integral O*1s* peak shows a good decomposition into three elementary A, B, and C peaks with E_b_(O*1s*)~529.7, 531.6, and 532.6 eV, respectively, with the positions shown in Table 3 for all samples. The peaks with E_b_(O*1s*)~529.7 eV relate to the lattice oxygen in iron oxides, while peaks with higher E_b_ values relate to oxygen states of surface layers in which coordination and oxygen state differ from oxygen location in the bulk phase of oxide. The E_b_ values of these oxygen states correspond to the surface hydroxyl and carbonate groups [49,50], which can result from the interaction of water vapors and CO_2_ with the reactive surface centers in the post-synthetic period.

Hydroxylation of the α-Fe_2_O_3_(0001) surface occurs at low (~10^−8^ torr) water vapor pressures, forming several types of hydroxyl groups as known from [50]. According to calculations made in [49], the chemisorption of water vapor on a defective surface, specifically on anion vacancies, is preferable. The formation of mono- and bidentate carbonate and bicarbonate groups occurs during the carbon dioxide adsorption on the hematite surface according to [50,51,52], and this formation proceeds more efficiently in the presence of hydroxyl groups [51,52].

On the surface of the studied α-Fe_2_O_3_ calcined samples, the proportion of out-of-lattice oxygen forms was estimated from the ratio of the sum of the areas of peaks B and C to the total O*1s* spectrum area (O(B) + O(C)/O_total_). Figure 7b shows that the proportion of out-of-lattice oxygen forms significantly increases from 0.27 to 0.37 with increasing calcination temperature of α-Fe_2_O_3_ samples. There is a correlation between the content of surface out-of-lattice oxygen forms and the content of Fe^2+^ ions well explained by the change in the proportion of removed lattice oxygen from α-Fe_2_O_3_, which increases with increasing calcination temperature. Here, the concentration of surface defect centers increases, in particular, of anion vacancies, which are centers of water adsorption, localized Fe^2+^ ions, and specific centers of CO_2_ adsorption. Some decrease in the ratio of O(B) + O(C)/O_total_ from 0.37 to 0.35 for the Fe_2_O_3_-1100 sample is likely due to partial ordering and crystallization of the surface phases.

Thus, investigating the surface of α-Fe_2_O_3_ samples calcined in the air in the temperature range of 800–1100 °C using the XPS method showed that the calcination temperature affects the surface chemical composition. With increasing calcination temperature, the surface enrichment with Fe^2+^ ions occurs, which agrees with the Raman results and the increase, compared with the stoichiometric value for the hematite phase, of the O/Fe ratio due to the significant contribution of the proportion of out-of-lattice (hydroxyl and carbonate) oxygen forms.

Based on the set of the obtained study results of α-Fe_2_O_3_ samples calcined in the interval of 800–1100 °C, the effect of high-temperature pretreatment on the morphological characteristics, chemical state of the surface, microstructural parameters, and the lattice oxygen reactivity regarding hydrogen was established.

According to X-ray diffraction data, all samples are represented by the α-Fe_2_O_3_ phase, in which crystal density value monotonically increases with increasing calcination temperature.

According to XPS results, on the surface of all α-Fe_2_O_3_ calcined samples, low-valent Fe^2+^ ions and out-of-lattice (hydroxyl and carbonate) forms of oxygen present, which proportion increases with increasing α-Fe_2_O_3_ calcination temperature.

According to Raman spectroscopy results, all samples consist of two types of particles represented by the α-Fe_2_O_3_ phase with differences in the size of crystallites, degree of crystallinity, and disorder of the crystal lattice. Small particles, consisting of crystallites with a lower degree of crystallinity, are on the surface of larger particles with a well-crystallized α-Fe_2_O_3_ phase. With increasing calcination temperature of α-Fe_2_O_3_ samples, the proportion of surface particles decreases, and their size increases. Traces of the magnetite phase are registered in the surface particles of the samples calcined at 1000–1100 °C. The crystal lattice of well-crystallized α-Fe_2_O_3_ crystallites of all the calcined samples has a disorder evidenced by activating the prohibited in the Raman spectra LO mode at 659 cm^−1^. For these crystallites, there is an inverse correlation between the intensity at the 226 cm^−1^ (A_1g_) line, which belongs to the vibrations of iron ions along the *c* crystallographic axis, and the unit cell parameter (*c*) calculated from X-ray diffraction data.

The observed differences in the structural features and the surface state of α-Fe_2_O_3_ samples calcined at 800–1100 °C make it possible to conclude that the main reason for the low reactivity of high-temperature α-Fe_2_O_3_ samples (Fe_2_O_3_-1000 and Fe_2_O_3_-1100) regarding hydrogen is due to the formation of α-Fe_2_O_3_ samples with a denser crystal lattice and with the surface enriched with Fe^2+^ iron ions, which deactivate Fe-O bond.

## 4. Conclusions

This paper studied the relationship between the textural and microstructural characteristics and the surface state of α-Fe_2_O_3_ samples calcined in the temperature range of 800–1100 °C with the activity of these samples regarding hydrogen in the TPR mode in the range of 40–900 °C.

It was found that the oxygen reactivity of α-Fe_2_O_3_ regarding hydrogen decreases with increasing calcination temperature of the samples.

All samples of hematite are monophase and represented by the α-Fe_2_O_3_ phase. With an increase in the calcination temperature from 800 to 1100 °C, the crystal density of the phase increases from 5.2704(1) to 5.2713(1) g/cm^3^.

According to the Raman spectroscopy results, all samples consist of two types of particles: large, with a well-crystallized α-Fe_2_O_3_ phase; and smaller particles, which are on the surface, also represented by the α-Fe_2_O_3_ phase with a significantly lower degree of crystallinity. Based on the registration of the line at 659 cm^−1^ (LO mode) prohibited in the Raman spectrum of α-Fe_2_O_3_, we can identify the presence of a close type of crystal lattice disorder in large particles of all calcined samples. There are traces of magnetite in small particles in the samples calcined at 1000–1100 °C.

According to the XPS results, on the surface of all calcined α-Fe_2_O_3_ samples, there are Fe^2+^ ions and out-of-lattice oxygen forms, whose concentrations increase with increasing calcination temperature of samples.

The study results indicate that by increasing the calcination temperature to 1100 °C, the α-Fe_2_O_3_ samples form a denser, less disordered crystal oxide lattice with more surface content of Fe^2+^ ions, which leads to an increase in the binding energy of oxygen (deactivation of the Fe-O bond) and decreased reactivity regarding hydrogen. These results can help develop catalytic systems and oxygen carriers based on α-Fe_2_O_3_ for high-temperature catalytic oxidation processes, cyclic processes of fuel combustion, hydrogen generation (CLC, CLGH), etc.

## Figures and Tables

**Table 1:** Specific surface area, pore volume, average particle size, crystal density of α-Fe_2_O_3,_ and the temperature values of the initial reduction and 95% reduction degree for calcined hematite samples.

T_cal_, °C	SSA, m^2^/g	V_pore_, × 10^−4^ cm^3^/g	D_av_, μm	*D_X_*, g/cm^3^	T_in._, °C	T_95%_, °C
800	2.10	27.4	~0.2	5.2704(1)	337	630
900	0.81	11.5	-	5.2707(1)	385	668
1000	0.21	3.1	-	5.2711(1)	429	737
1100	0.09	1.6	~2.5	5.2713(1)	435	812

V_pore_ is the total pore volume at P/P_0_ = 0.95, D_av_ is the average size of crystal grains from SEM image analysis, and D_X_ is the density of α-Fe_2_O_3_ from X-ray diffraction data.

**Table 2:** The concentration of elements in calcined hematite samples (800–1100 °C), the ratios of elements are normalized for iron content.

Samples	Concentration of Elements, Atomic Ratios				
	C_Σ_	C(CO_3_)	O_Σ_	Fe	Si *
Fe_2_O_3_-800	1.21	0.04	1.52	1	0.08
Fe_2_O_3_-900	1.77	0.13	1.82	1	0.24
Fe_2_O_3_-1000	2.23	0.17	2.10	1	0.21
Fe_2_O_3_-1100	1.59	0.14	1.89	1	0.10

* Si is not registered in the sample of the initial hematite reagent, so in the calcined samples, the presence of Si is due to the procedure of grinding sintered samples in the mill to obtain the particle-size fraction.

**Table 3:** Peak positions of the XPS lines Fe*2p*, O*1s,* and C*1s* (E_b_ in eV).

Samples	Fe*2p_3_*_/*2*_				O*1s*			C*1s*		
	Fe^2+^		Fe^3+^		A	B	C	A	B	C
Fe_2_O_3_-800	709.9	714.1	711.5	719.0	529.7	531.6	532.6	285.0	286	288.5
Fe_2_O_3_-800	709.9	714.1	711.4	710.0	529.7	531.6	532.5	285.0	-	288.6
Fe_2_O_3_-800	709.9	714.1	711.5	718.8	529.7	531.6	532.7	284.9	-	288.6
Fe_2_O_3_-1100	709.8	714.1	711.6	718.9	529.6	531.5	532.7	285.0	-	288.7

## Data Availability

Not applicable.
